# Enhancing ECG-based heart age: impact of acquisition parameters and generalization strategies for varying signal morphologies and corruptions

**DOI:** 10.3389/fcvm.2024.1424585

**Published:** 2024-07-04

**Authors:** Mohammed Yusuf Ansari, Marwa Qaraqe, Raffaella Righetti, Erchin Serpedin, Khalid Qaraqe

**Affiliations:** ^1^ Electrical and Computer Engineering, Texas A&M University, College Station, TX, United States; ^2^ Electrical and Computer Engineering, Texas A&M University at Qatar, Doha, Qatar; ^3^ Division of Information and Computing Technology, College of Science and Engineering, Hamad Bin Khalifa University, Qatar Foundation, Doha, Qatar

**Keywords:** ECG age estimation, ECG acquisition, ECG sampling duration, ECG sampling rate, deep learning, neural network, ECG waveform variability, ECG distortion

## Abstract

Electrocardiogram (ECG) is a non-invasive approach to capture the overall electrical activity produced by the contraction and relaxation of the cardiac muscles. It has been established in the literature that the difference between ECG-derived age and chronological age represents a general measure of cardiovascular health. Elevated ECG-derived age strongly correlates with cardiovascular conditions (e.g., atherosclerotic cardiovascular disease). However, the neural networks for ECG age estimation are yet to be thoroughly evaluated from the perspective of ECG acquisition parameters. Additionally, deep learning systems for ECG analysis encounter challenges in generalizing across diverse ECG morphologies in various ethnic groups and are susceptible to errors with signals that exhibit random or systematic distortions To address these challenges, we perform a comprehensive empirical study to determine the threshold for the sampling rate and duration of ECG signals while considering their impact on the computational cost of the neural networks. To tackle the concern of ECG waveform variability in different populations, we evaluate the feasibility of utilizing pre-trained and fine-tuned networks to estimate ECG age in different ethnic groups. Additionally, we empirically demonstrate that finetuning is an environmentally sustainable way to train neural networks, and it significantly decreases the ECG instances required (by more than 100×) for attaining performance similar to the networks trained from random weight initialization on a complete dataset. Finally, we systematically evaluate augmentation schemes for ECG signals in the context of age estimation and introduce a random cropping scheme that provides best-in-class performance while using shorter-duration ECG signals. The results also show that random cropping enables the networks to perform well with systematic and random ECG signal corruptions.

## Introduction

An electrocardiogram (ECG) is the gold-standard modality used to capture the overall electrical activity produced by the contraction and relaxation of the cardiac muscles. Its core aim is to capture cardiac function and abnormalities (1–7). Electrophysiologists analyze the ECG by extracting medically established parameters (e.g., heart rate variability (HRV), duration length of PR, QRS, and ST intervals, etc,) and compare them with clinical guidelines to make an accurate diagnosis. However, the ECG interpretation process is subjective, requires highly trained experts, and is prone to errors due to its time-consuming nature. In recent years, deep learning has revolutionized the field of computer vision, medical imaging, and drug discovery (8–19) due to its ability of automatic feature extraction. Specifically, convolutional neural networks (CNNs) (20) have helped overcome the challenge of subjective ECG interpretation by learning the patterns associated with the cardiac condition (21). A multitude of innovative deep-learning architectures have been trained on large-scale ECG data to detect irregular heart rhythms (22–24), abnormal cardiac pulsations (25–27), and incidents of myocardial infarction (i.e., heart attack) (28–30). However, deep learning methodologies typically focus on optimizing performance for a single diagnostic task, such as detecting arrhythmias or predicting myocardial infarctions. This approach leads to a lack of efficient solutions that can analyze twelve-lead ECG signals for multiple diseases and provide a comprehensive overview of cardiac health. The primary reasons for this gap are the lack of datasets containing diverse cardiac disease ground truths for the same ECG signals and the performance degradation of neural networks when retrained for new tasks. Therefore, developing a quantifiable metric to assess overall cardiovascular health would be of great clinical significance.

ECG signals can be utilized to derive a metric of overall cardiovascular well-being because they capture a fingerprint of many biological processes occurring within the human body. To elaborate, it has been shown that long-term mental stress, anxiety, and chronic depression (31, 32) can have a significant impact on heart rate and HRV, thereby modifying the ECG signal waveform and imprinting the presence of mental distress in the ECG signals (33, 34). Furthermore, using the UK Biobank database, Verwiej et al. (35) empirically proved that twelve-lead ECG signals are correlated with 300 genetic loci. ECG signal amplitudes and waveform duration evolve with healthy aging. Concurrently, ECG signal waveforms are also affected by latent cardiovascular factors (i.e., cardiac diseases and structural changes). Given the genetic underpinning, regular aging, and latent cardiovascular factors influencing the ECG signals, ECG can be utilized to derive complex metrics such as age. Specifically, ECG-derived age (ECG age) using deep neural networks can be used as a measure of cardiovascular well-being (36, 37). Neural networks are ideal for this task because of their capability to learn patterns associated with age-related waveform changes and latent cardiovascular factors, such as cardiovascular diseases (CVDs). This allows the networks to associate with each patient an estimate (i.e., ECG age), which is close to the chronological age for healthy individuals but is higher than the patient’s chronological age for individuals with CVDs. In literature, it has been established that a large positive difference between estimated ECG age and chronological age (i.e., delta age) is associated with several cardiovascular disorders (38, 39), CVD risk factors (40), cardiac anomalies, and a higher mortality rate (41). By evaluating overall cardiovascular well-being using ECG age/delta age, medical professionals can track the cardiac health of patients who do not exhibit positive results through conventional diagnostic tests for CVDs but display preliminary symptoms, leading to effective patient management while limiting disease progression.

Several deep learning-based (42–44) methodologies have been proposed to estimate ECG age from twelve-lead ECG signals effectively. The existing studies can be stratified as technical or applied based on the objective of the methodology. The technical studies propose one-dimensional (1-D) neural networks for estimating ECG age and highlight its significance in a new population race. On the other hand, applied studies utilize the pre-trained network proposed in the technical studies to establish an association of ECG age with CVDs, cardiac anomalies, higher mortality rate, etc. Attia et al. (37) propose a simple 1-D CNN to estimate ECG age using raw twelve-lead ECG signals. The study details an architecture that contains eight convolutional blocks for capturing the temporal features and one convolutional block for modeling the spatial features. Additionally, the authors studied the changes in the ECG age for a cohort of 100 patients to gain insight into the variations with the disease diagnosis over twenty years. Lima et al. (45) suggest the use of a relatively deeper 1-D residual neural network to predict the ECG age for the Brazilian population. The network contains a convolutional block for primary feature extraction followed by four residual blocks for advanced feature extraction without loss of temporal information due to max-pooling. Empirical results from this study highlight that ECG age can be a statistically significant predictor of CVD risk. Chang et al. (40) utilize an ECG12Net architecture, originally designed for detecting Hypokalemia and Hyperkalemia from ECG signals, for estimating ECG age. The authors employed Class Activation Mapping (CAM) techniques to visualize the regions of the signals highly emphasized by the network.

The outcomes of the technical studies (i.e., pre-trained neural networks) have been employed in the applied studies to estimate ECG age in the sick and healthy population segments to establish an association of ECG age with CVDs and cardiac anomalies. Ladejobi et al. (46) utilize the AttiaNet to estimate the ECG age of primary care outpatients who had no cardiovascular diseases. The statistical results of the study showed that delta age ≥1 standard deviation (i.e., SD) had higher CVD and all-cause mortality, than delta age ≤1 SD, suggesting that ECG can serve as an independent predictor of all-cause and cardiovascular mortality. Toya et al. (39) employ AttiaNet to establish an association between ECG age and vascular aging by highlighting a strong positive correlation between large positive delta age and peripheral microvascular endothelial function (PMEF). The results specify that PMEF accompanied by large positive delta age increases the chances of adverse cardiovascular events. Benavente et al. (47) estimate the ECG age of Russian and Norwegian populations using AttiaNet to analyze the mortality rate in the two countries. The analysis indicates that increased delta age in the Russian population compared to the Norwegian population is in alignment with higher CVD mortality rates in Russia. Wall et al. (48) employ a simple multi-layer perceptron (MLP) trained using 577 ECG features of healthy volunteers to estimate ECG age. The authors improve the effectiveness of neural network training and interpretability by utilizing the Synthetic Minority Oversampling Technique (SMOTE) for balancing the dataset and SHapley Additive exPlanations (SHAP) for computing feature importance. The discussion emphasized that ECG age can exhibit a reversal of aging with effective treatment and timely patient care. Even though these applied studies successfully establish associations between ECG age and cardiovascular disorders, the strength association could be increased by improving the network performance through modifying and tuning the network architecture and employing robust loss functions and training routines.

A thorough analysis of the current studies reveals four significant limitations, with two stemming from the viewpoint of ECG data acquisition and the other two from the standpoint of ECG waveform variability and lack of robustness to signal distortions. The limitations due to ECG data acquisition arise because existing work ignores the impact of the sampling rate and signal duration on the predictive ability of neural networks. The sampling rate determines the density of information carried in a fixed duration of signal (e.g., a 100 Hz signal contains 100 information points within a 1-s duration), thereby impacting the morphology of the signals (critically at peak R amplitude). Similarly, signal duration is another parameter that determines the amount of temporal information carried by the signals, and greater temporal knowledge can allow the network to detect changes in the heart rate (i.e., HRV), abnormal heartbeats, and rhythms. Hence, it is imperative to ascertain whether the neural network’s performance for ECG age estimation is subject to variations due to different sampling rates and signal duration during training.

Another limitation of deep learning systems for ECG analysis is their insufficient robustness to variations in ECG morphology across different ethnic populations and their susceptibility to systematic and random signal corruptions. Data augmentation has been heavily employed in mainstream computer vision (e.g., horizontal and vertical flipping) and natural language processing (e.g., synonym replacement) to tackle the challenge of overfitting by increasing the effective dataset size. For instance, by randomly flipping the ECG signals across the zero-volt baseline or reversing the ECG signal, the effective dataset size can be increased to 4× the original data set size. Consequently, augmentation techniques have improved neural network performance in medical imaging (49), drug discovery (50), and speech recognition (51). Similarly, ECG augmentation may assist neural networks in generalizing effectively for small ethnic populations having limited datasets due to a lack of subjects or digital transformation. Furthermore, augmentation schemes can increase instances corresponding to rare CVDs, allowing networks to learn patterns corresponding to rare disorders. However, data augmentation techniques have not been employed in studies estimating ECG age and coping with signal corruptions and distortions. Therefore, it is important to determine the impact of different data augmentation routines on the age estimation performance of the network. Finally, a significant limitation of existing methodologies is the insufficient evaluation of network generalizability across diverse ethnic populations. Hence, it is imperative to determine strategies for neural network models trained on large ECG datasets for particular racial populations that can be utilized to estimate ECG age for different ethnic groups.

In light of these shortcomings in the literature, this paper conducts an empirical study on the AttiaNet (37) and ResNet1D (45) architectures that have been popular in the domain for estimating ECG age and establishing its clinical significance. Fundamentally, the article has the following key contributions:

1. We analyze the impact of the sampling rate on neural networks by training the networks at five different data acquisition rates and comparing their performance to establish a tradeoff between age estimation performance and signal information density. In addition, we analyze the impact in terms of computational cost, RAM, GPU memory utilization, and storage space requirement for networks across sampling rates and signal duration.
2. We investigate the degradation in the age estimation performance with the decrease in temporal information to determine a threshold for the signal duration that yields acceptable performance. Furthermore, we examine whether higher sampling rates can compensate for the loss of temporal information in the signals.
3. We examine the effect of different ECG augmentation techniques on network performance and suggest a novel augmentation routine for ECG age estimation. Additionally, we explore whether data augmentation could compensate for the performance loss due to shorter signal duration or corruption in ECG morphology.
4. We evaluate the feasibility of transfer learning and fine-tuning as strategies for generalizing across diverse signal morphologies. Additionally, we analyze the network’s performance by fine tuning it with datasets of varying sizes. Finally, we compare the training time and GPU power consumption of the fine-tuned networks and networks trained from scratch to showcase an environmentally sustainable pathway for ECG age estimation.

To the best of our knowledge, this study fills critical gaps in the literature and allows medical practitioners to use neural networks to estimate ECG age across diverse sampling rates, signal lengths, and racial populations encountered at large hospitals with high racial and ethnic diversity. Furthermore, the study serves as a valuable resource for fellow researchers in this field, clarifying ambiguities and imparting insights, such as the pivotal influence of data acquisition variables on network performance. This, in turn, empowers researchers to make well-informed and judicious decisions for their future methodologies.

The subsequent sections of the manuscript are structured as follows: Section “Neural network architectures and empirical methodology” provides a comprehensive account of the empirical methodology, encompassing an overview of neural network architectures, experiment design, and underlying empirical objectives. Section “Experimental setup” elucidates the experimental setup detailing the dataset, neural network training and evaluation routine, evaluation metrics, and implementation details that facilitate the reproducibility of the results. Section “Results and discussion” highlights the principal outcomes of the empirical study and discusses their implication on deep learning methodology design and prospective clinical applications for ECG age estimation. Section “Conclusion” provides a synthesis of our findings and concludes the article.

## Neural network architectures and empirical methodology

One-dimensional CNNs are popular for estimating age from twelve-lead ECG signals. Early convolutional layers detect amplitude changes, while deeper layers identify complex patterns like the QRS waveform. Advantages of convolution kernels over fully connected networks include shared kernel weights across the signal and the ability to combine information from multiple leads. These benefits make one-dimensional CNNs effective at extracting relevant features from ECG signals. Convolutional features are stacked along the channel dimension, and pooling operations reduce the temporal dimension while capturing salient features. The resulting dense feature map is processed by a multi-layer perceptron for ECG age estimation. For our preliminary study, we trained and evaluated the ECG12Net architecture (40) adopted for the ECG age estimation in the Taiwanese population. Subsequently, we utilized the widely adopted and commonly used architectures for ECG age estimation to conduct this study. A summary of technical terms pertaining to neural networks and their explanation is provided in the Glossary.

### AttiaNet architecture

Attia et al. (37) propose a one-dimensional neural network using convolutional layers in temporal and spatial domains for effective ECG feature extraction. The network includes eight temporal convolutional blocks followed by a spatial convolutional block for feature fusion across all leads. Each block consists of a convolutional layer with ReLU activation, batch normalization for stabilization and generalization, and max-pooling for dimension reduction. Kernel sizes are adjusted across layers: seven for the initial block, five for intermediate layers, and three for deeper layers, enabling adjustment in the receptive field of the network as the feature map dimension decreases. Mathematically, the fundamental convolutional building blocks of the AttiaNet architecture can be expressed as follows:

(1) $$\;\begin{array}{cc} & Conv_{\_m}(x,\;K;\theta )=(w^{i}⊛x+b^{i}),\;\forall 1\leq i\leq K,\;w^{i}\in \theta ,\;b^{i}\in \theta \\ & Conv\_batchnorm_{\_m}(x,\;K;\theta )=ReLU(BN(Conv_{\_m}(x,\;K;\theta )))\\ & \;Conv\_Block_{\_m}(x,\;K,\;P;\theta )=\\ & \;Maxpool(Conv\_batchnorm_{\_m}(x,\;K;\theta ),\;P),\; \end{array}$$

where the symbol x stands for the input feature map, K represents the number of convolutional kernels, m is the convolutional kernel size, and P denotes the size of maxpool window; θ encapsulates the weights and biases of all convolutional kernels. Operator ⊛ defines the one-dimensional convolution operation, $w^{i}$ and $b^{i}$ represent the weight and bias of the ith convolutional kernel, BN indicates the batch normalization operation applied to the output of convolution, and ReLU is the activation function that introduces non-linearity in the network (Eq. 1).

### ResNet1D architecture

**Figure 1:**
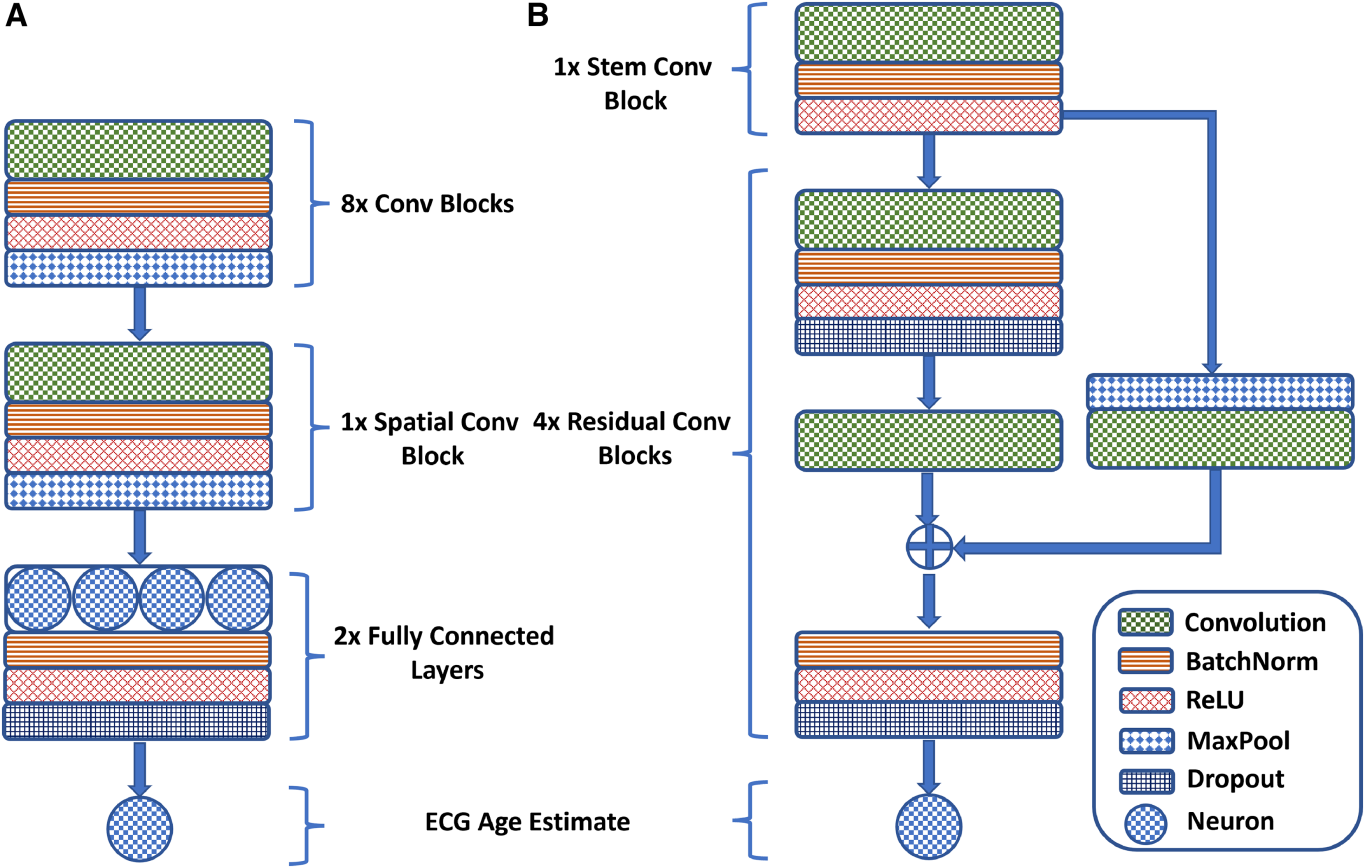
A visual overview of the (**A**) AttiaNet and (**B**) ResNet1D architectures in terms of their fundamental building blocks.

Lima et al. (45) adapt the ResNet1D architecture to estimate ECG age in a Brazilian cohort. Initially, a convolutional block, similar to AttiaNet, extracts preliminary features, which are then processed through four residual blocks for advanced feature extraction. The network uses a large kernel size of 17 to capture distant temporal relations. The residual blocks reduce the temporal dimension by a factor of 4 while increasing the channel dimension by 64. Skip connections are included to minimize information loss and address vanishing gradients, incorporating max-pooling and 1×1 convolutions to align feature map dimensions. The mainstream network pathway of the residual blocks comprises two convolutional layers, with each convolution followed by a dropout layer to reduce potential overfitting. Furthermore, the skip connection is merged with the mainstream before the final activation, batch normalization, and dropout to ensure that the output of the residual blocks is normalized. Mathematically, the core residual blocks of the ResNet1D architecture implement the following operations:

(2) $$\begin{array}{cc} & Skip\_Path=Conv_{\_1}(Maxpool(x,\;P),\;C;\theta_{s})\\ & Main\_Conv\_1=Dropout(Conv\_batchnorm_{\_17}(x,\;C;\theta_{1}))\\ & Main\_Conv\_2=Conv_{\_17}(Main\_Conv\_1,\;C;\theta_{2})\\ & Resblock\_Output=Dropout(ReLU(BN(Main\_Conv\_2\\ & +Skip\_Path)))),\; \end{array}$$

where the symbol x is the input to the residual block, C indicates the number of convolutional kernels, P represents the temporal pooling window length, $\theta_{1}$ and $\theta_{2}$ are the main stem convolution kernel parameters, and $\theta_{s}$ is the 1×1 convolutional kernel parameter for the skip-connection. Figure 1 provides a visual overview of the design of the AttiaNet and ResNet1D architectures in terms of the core convolutional and residual blocks (Eq. 2).

### Empirical methodology design

This subsection describes the motivation, objectives, and details of the experiments that are part of this empirical investigation.

#### Sampling rate

One of the key data acquisition parameters for ECG signals is the sampling rate. ECG sampling rate determines the information density in a signal within a fixed duration. The sampling rate parameter must be carefully tuned in the ECG data collection protocol because a sub-optimal sampling rate may provide savings in terms of storage space but can negatively impact the signal morphology and introduce artifacts due to the loss of high-frequency information (i.e., aliasing). Conversely, ECG signals captured at a very high sampling rate may have excessive storage requirements, signal noise, and limited performance benefits, making the ECG analysis challenging. As a result, the ECG sampling rate parameter should be thoroughly examined to assist the researchers in building cutting-edge deep learning architectures. In order to achieve this objective and ensure the applicability of our results across diverse neural network architectures, we employ the AttiaNet and ResNet1D models to train on 10-s twelve-lead ECG data at both 100 Hz and 500 Hz sampling rates. Subsequently, we compare the model performance difference between the two sampling rates considering the network training time, memory utilization, and inference time to comprehensively evaluate the impact of the sampling rate at computation cost.

#### Signal duration

Similarly, ECG signal duration is another parameter that needs to be carefully selected in the data collection protocol for providing sufficient temporal information to electrophysiologists for making effective disease diagnoses. The performance of the neural network architecture for ECG age estimation may vary based on the extent of temporal information provided to the model as input. Longer signal duration encompasses electric impulse information of multiple cardiac cycles, allowing the networks to adjust the ECG age for abnormalities like abnormal HRV and arrhythmia. However, longer ECG signals require a larger storage budget and computation resources, thereby limiting the widescale adoption of ECG on consumer machines and mobile devices. In light of this tradeoff between network performance and computation/storage requirements, we train AttiaNet and ResNet1D architectures across five different signal durations (i.e., 2, 4, 6, 8, and 10 s) to establish a recommended ECG signal length for acceptable ECG age estimation performance while accounting for network training time, memory utilization, and inference time. We conduct these experiments using the 500 Hz sampling rate to minimize the impact of the sampling rate on the performance of the networks. Additionally, we analyze whether upsampling short ECG signals could partially compensate for the loss of temporal information for the ECG age estimation task.

#### Data augmentation and signal corruption

Along with the data acquisition parameters, data preprocessing and data set size (i.e., ECG instances) can significantly impact the neural networks’ performance. Data augmentation schemes are popular in mainstream computer vision and natural language processing to tackle the data-demanding nature of the networks. These schemes generate additional data instances by applying transformations or modifications to existing data while retaining the essential characteristics and structure of the original data, thereby increasing the effective size of the data, minimizing overfitting, improving the generalizability, and increasing the robustness to unseen data. Consequently, designing an effective data augmentation scheme for ECG age estimation could allow for significant improvement in the existing networks’ performance or equivalent performance with a smaller data set size. To determine the augmentation’s impact and identify the best-performing scheme, we train both AttiaNet and ResNet1D architectures by individually applying reverse, flip, and reverse&flip augmentations to every training batch with a certain fixed probability. Additionally, we analyze the effect of training the network with arbitrary random crops of ECG signal (say, k-s) out of the 10-s original signal (sampling rate of 500 Hz), thereby evaluating the performance uplift for ECG age estimation with shorter duration ECG signals. Finally, we investigate whether systemic elimination or corruption of certain ECG components (e.g., QRS transform, T-wave) due to technical issues in ECG machines or random loss of signal information due to poor contact of electrodes can degrade the performance of neural networks. Subsequently, we examine whether random crop augmentation schemes can compensate for the loss of signal information and allow networks to provide acceptable performance with corrupted ECG signals. For this final component of the study, we only utilize the AttiaNet model due to the computationally heavy nature and long training times of the ResNet1D architecture.

#### Transfer learning

**Figure 2:**
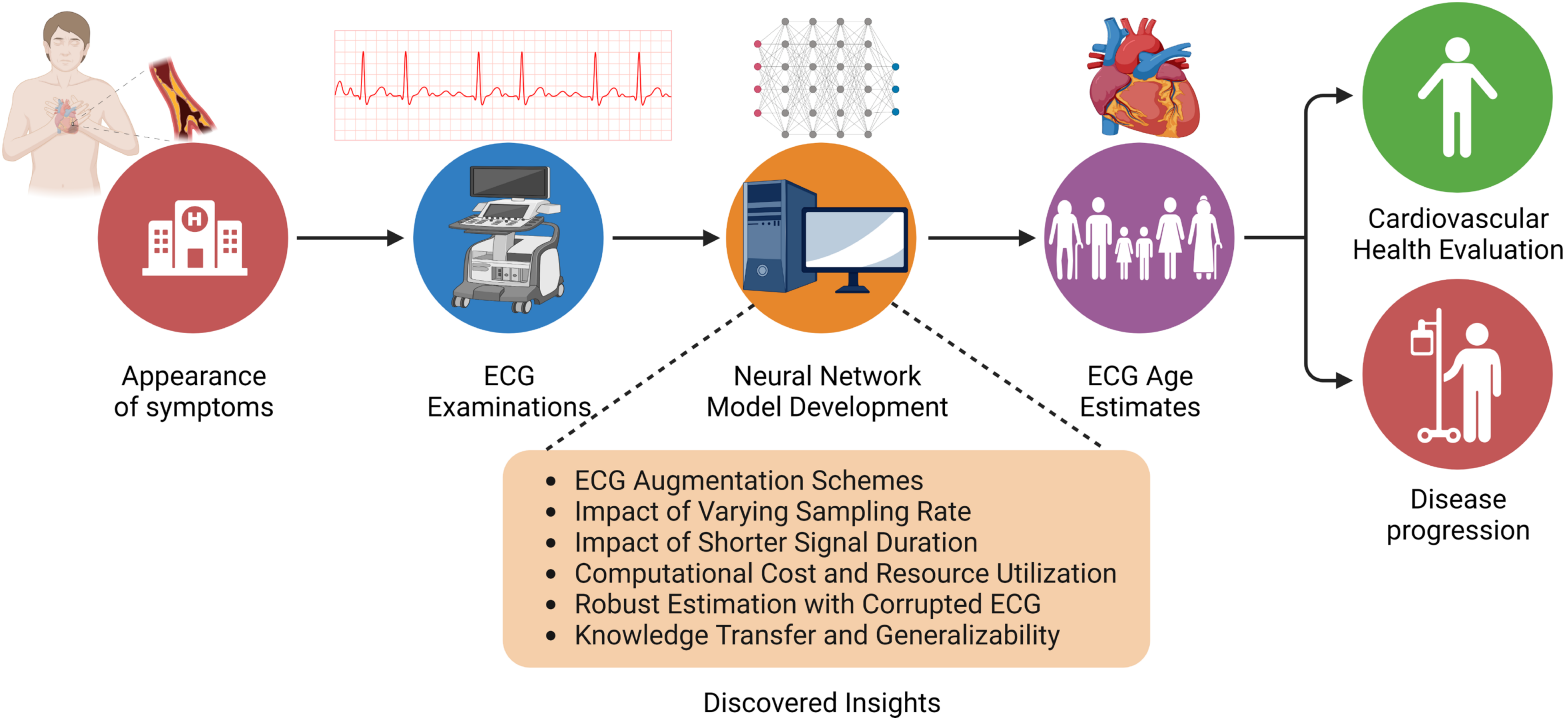
Overview of the ECG age estimation workflow, highlighting the insights gained from this empirical study.

A key factor that determines the wide-scale adaptability of neural network-based methodologies is their ability to generalize on a diverse variety of unseen data. Network generalizability over diverse ethnic populations is critical in the case of ECG-driven age estimation and diagnostic tools due to a well-established correlation between genetic loci and ECG signals (35). Although prior studies of ECG age estimation have verified the network’s performance on holdout sets drawn from the same data source (37) or with external validation sets from the same geographical region (45), it is essential to establish whether existing pre-trained models can be employed to estimate ECG age in a completely different ethnic population. To meet this objective, we evaluate the performance of the ResNet1D architecture with pre-trained weights from the CODE cohort study on the PTB-XL dataset (52). In this empirical investigation, we are compelled to restrict our experiments to the ResNet1D architecture because pre-trained weights of AttiaNet on the Mayo Clinic ECG dataset are not publicly available. One key advantage of neural networks over conventional machine learning methodology is their ability to transfer knowledge (i.e., transfer learning) between similar tasks while working with the same modality of input. We hypothesize that the ResNet1D architecture with pre-trained weights from the CODE cohort study can be fine-tuned to maximize the performance on the PTB-XL dataset or match the performance with a significantly smaller portion of training data. We assess the efficacy of transfer learning in the context of ECG age estimation by fine-tuning the network with uniformly sampled fine-tuning training sets of varying sizes. Additionally, we compare the performance attained by the ResNet1D model with fine-tuning and scratch training with small training set sizes, thereby emphasizing the significance of transfer learning and fine-tuning for developing models which could generalize over different ethnic populations with limited ECG data. Figure 2 shows the workflow of the ECG age estimation pipeline and the key objectives of this study.

## Experimental setup

In this section, we present vital information about the dataset for neural network training, the ECG augmentation strategies and their protocols, and the implementation specifics of the experiments performed to facilitate the reproducibility of this empirical investigation.

### Datasets and augmentation schemes

The PTB-XL (52) dataset comprises 21,799, 10-s twelve-lead ECG records of 18,869 patients, obtained using ECG machines from Schiller AG over seven years (October 1989–June 1996). Two cardiologists have stratified the ECGs in the dataset into five superclasses. The metadata of PTB-XL also provides a *fold_id* for a 10-fold cross-validation split that accounts for patient-level splitting, meaning all records of a particular patient are assigned to the same fold. Table 1 presents the clinical characteristics of the PTB-XL dataset. For the transfer learning study, we utilize the weights of ResNet1D architecture trained using the Clinical Outcomes in Digital Electrocardiography (CODE) cohort developed by the TeleHealth Network of Minas Gerais (TNMG), Brazil. The CODE cohort contains a total of 1,558,415 patients with a mean age of 51.6±17.6 years and 40.2% male.

**Table 1:** Clinical characteristics of the PTB-XL (52) dataset.

Characteristic	Description
Total ECG Records	21,799
Total Patients	18,869
Gender Distribution	52% male, 48% female
Age Range	0 to 95 years (Median: 62 years, Interquartile Range: 22 years).
Diagnosis Label Distribution	- Normal ECG (NORM): 9,514 records - Myocardial Infarction (MI): 5,469 records - ST/T Change (STTC): 5,235 records - Conduction Disturbance (CD): 4,898 records - Hypertrophy (HYP): 2,649 records
ECG Statement Annotations	Annotated by up to two cardiologists; 71 different ECG statements conforming to the SCP-ECG standard (diagnostic, form, rhythm).
Data Acquisition	Standard set of 12 leads with reference electrodes on the right arm. Metadata on age, sex, weight, height. Annotations with SCP-ECG statements, heart axis, infarction stadium.
Data Validation	Large fraction of records validated by a second cardiologist and a technical expert focusing on signal characteristics.
Data Preprocessing	Personal information pseudonymized. Age recorded in compliance with HIPAA standards.
File Format	WaveForm DataBase (WFDB) format. 16-bit precision at 1μV/LSB resolution.

In our study, we employ the *ecg_augmentation*^1^ package written in Python to examine the impact of various augmentations on the performance of the networks. During training, each augmentation (i.e., reverse, flip, and reverse&flip) is applied with a probability of 0.5 to a training batch, allowing the network to train on the transformed and original signals with equal likelihood. In contrast, random crop augmentation is applied with a 100% likelihood to each batch, resulting in a selection of a k-s patch with an arbitrary starting point that lies in the range of [0, 10-k]. Random crop augmentation significantly increases the dataset size by feeding the network with a different segment of the 10-s signal at every epoch. We ensure that these augmentations are applied across the twelve-lead, thereby ensuring that the network receives a temporally correct and consistent order of information from all leads. On the other hand, the experiments evaluating the impact of corrupted signals employ feature masks (i.e., PR, QRS, and QT masks) or random masks, which are applied with a probability of 0.25 to each lead. Independent application of masks to the ECG leads forces the networks to utilize the information in uncorrupted leads, thereby preventing the network from relying on particular ECG leads.

### Implementation details

The study utilized Python to evaluate the impact of data acquisition parameters, network training, and generalizability. The networks are implemented using PyTorch (version 1.9),^2^ while the utility functions are provided by Pandas, Scipy, and Matplotlib. Specifically, the AttiaNet is implemented from scratch using the details provided in the manuscript. The source code and pre-trained weights of ResNet1D architecture are obtained from the open-source GitHub repository.^3^ Before initiating the experiments, the WFDB files for 100 Hz and 500 Hz ECG signals in the PTB-XL dataset are converted into NumPy arrays and loaded into the system memory to minimize the I/O during network training and evaluation, thereby minimizing disk load and seek times. The Numpy arrays are fed to a custom PyTorch Dataset module to apply augmentations with a fixed probability to training batches. PyTorch Datasets are forwarded to PyTorch Dataloader to generate data batches using multiple CPU cores, thereby maximizing GPU utilization during training and minimizing training times. Mean square error is used as a loss function to quantify the discrepancy between the network-generated ECG age and the chronological age. Subsequently, the Adam optimizer is utilized to update the network weights based on the loss function. The networks are trained for 200 epochs with a batch size of 96 and an initial learning rate of 0.001. A learning rate scheduler decreases the learning rate by a factor of 10 after every 80 epochs, allowing the networks to converge effectively. During the training, the network weights are saved for epochs that result in a minimum mean square error on the holdout validation set. To robustly evaluate the objective of our experiments, the networks are trained with 10-fold cross-validation labels provided in the PTB-XL dataset, and the best weights are saved for every fold. The provided test errors in the results section are the average of the 10-fold, thereby adding to the confidence and robustness of the outcomes of this empirical investigation.

The details pertaining to the individual experiments are as follows. In order to investigate the impact of data acquisition parameters, intermediate sampling rates (i.e., 200, 300, and 400 Hz) are generated by applying interpolation schemes on the 100 Hz ECG signals. To determine the impact of temporal duration on the network performance, the first k-s (i.e., k=2,4,6, and 8) of the 10-s signal are used to train, validate, and test the neural networks. To evaluate the generalizability of the pre-trained network, we use ResNet1D architecture with CODE cohort weights during the network evaluation. To finetune the ResNet1D architecture, the convolutional layers until the third residual block are frozen, thereby allowing the training of the fourth residual block and fully connected layer.

Neural network training and evaluation are performed on a supercomputing cluster provided by Texas A&M High-Performance Research Computing. Specifically, a node comprising 16 CPU cores, 64 GB of system memory, and an Nvidia T4 GPU with 16 GB of VRAM is utilized for running the experiments.

### Evaluation metrics

We evaluate the performance of the ECG age estimation networks with the following well-known metrics for regression:

• **Mean square error (MSE):** The error is computed by taking the square of the difference of the ECG estimated age and chronological age averaged across all the instances in the dataset. MSE is often used in our experiments to compare the accuracy of different networks trained with varying data acquisition parameters and augmentation schemes.
• **Mean absolute error (MAE):** The error is computed as the absolute difference between ECG age and chronological age. MAE is preferred to interpret the variations in ECG age estimated by the networks.
• **Training and inference time:** The training time per epoch and inference time for the entire test set is reported in seconds to compare the operational efficiency of the neural networks both during the re-training phase and deployment.
• **Disk and VRAM utilization:** Storage requirement for storing the the neural network parameters along with VRAM utilization is reported in MBs to clarify the hardware requirements for the training and deployment of the networks.
• **Parameter count and multiplications-additions:** Number of trainable and non-trainable weights of the networks along with the count of multiplications and additions is reported to measure the computational complexity.

## Results and discussion

In this section, we present the results of the four empirical investigations and discuss their impact on future deep learning-based methodologies for ECG age estimation. Our preliminary experiments with ECG12Net architecture proposed for the Taiwanese population resulted in a test MSE of 117.14 on the PTB-XL dataset. The limited performance of the ECG12Net architecture is due to overfitting caused by a large number of parameters in the network backbone. We observed the ResNet1D architecture has relatively fewer parameter and has been robustly evaluated on multiple ECG datasets for the South American population. Consequently, for this empirical study, we have utilized AttiaNet and ResNet1D architectures over the ECG12Net due to their superior age estimation performance.

### Impact of sampling rate

**Figure 3:**
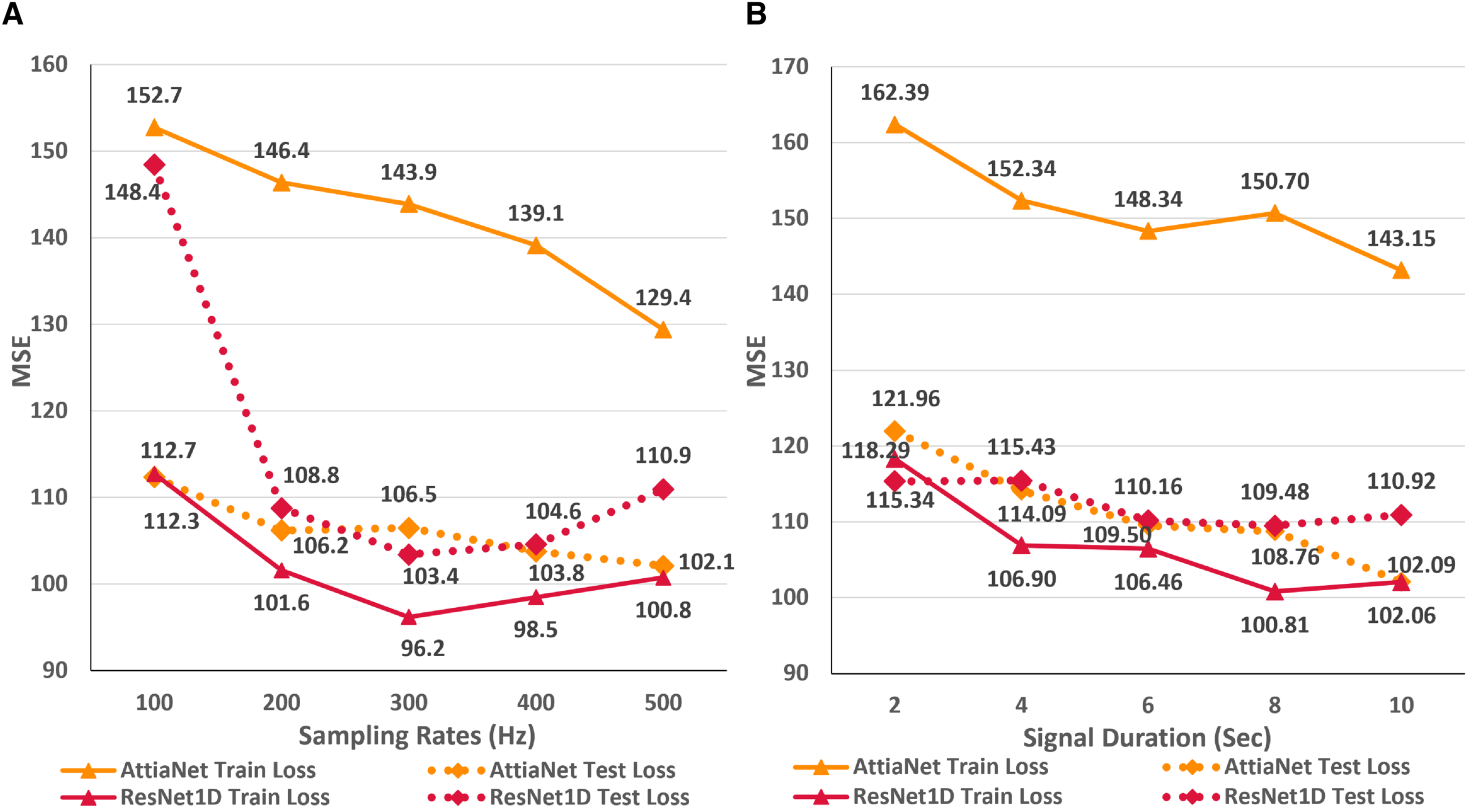
(**A**) Line plot highlighting the trends in train and test MSE for the AttiaNet and ResNet1D architecture across the five different sampling rates. (**B**) Line plot highlighting the trends in train and test MSE for the AttiaNet and ResNet1D at 500 Hz when trained with signals of different lengths.

In this first empirical study, we evaluate the impact of ECG sampling rate on the performance of the neural networks considering computational cost, VRAM and disk utilization, and inference times. Figure 3A presents the variations in the train and test MSE for the AttiaNet and ResNet1D architectures across sampling rates ranging from 100 Hz to 500 Hz. A high-level analysis of the line plot indicates that both train and test MSE show an overall decreasing trend as the sampling rate increases from 100 Hz to 500 Hz. For AttiaNet architecture, train MSE decreases from 152.7 to 129.4 as the sampling rate increases. However, the train MSE remains higher than the test MSE, suggesting a potential underfitting due to a simplistic 1D CNN design of the AttiaNet architecture. The dotted line highlights that AttiaNet experiences a sharp drop in MSE (i.e., improvement in age estimation) as the sampling rate increases from 100 Hz to 200 Hz, followed by a plateau at 300 Hz and a gradual decrease at higher sampling rates. Overall, as the sampling rate increases from 100 Hz to 500 Hz, the test MSE decreases from 112.7 to 102.1, resulting in a 9.4% improvement in performance.

For ResNet1D architecture, train MSE remains below the test MSE for all sampling rates, thereby overcoming the issue of underfitting experienced by the AttiaNet architecture. At 100 Hz, the significant gap between the train and test MSE for the ResNet1D architecture implies that the network is overfitting the data due to low information density (1,024 data points) and high parameter count (6.94 million). Increasing the sampling rate, the test MSE decreases sharply from 148.4 to 108.8 (26.6% improvement) at 200 Hz and reaches a minimum of 103.4 (30.3% improvement) at 300 Hz. Interestingly, the performance of the ResNet1D architectures diverges from the minima as the sampling rate increases further to 500 Hz. This divergence in the performance is due to the design of the ResNet1D top, which directly connects the output of the 1D convolution to the linear output unit. As the sampling rate increases by 100 Hz, the data points in the signal grow by 1,024 units, resulting in increasingly longer feature maps at 400 Hz and 500 Hz. After the final convolutional layer, the flattened feature maps are directly connected to the output neuron, creating an information bottleneck, which results in performance degradation. Nevertheless, AttiaNet and ResNet1D architectures benefit significantly from the higher sampling rates between 300 and 500 Hz.

Tables 2 and 3 provide an overview of several important measures for evaluating the AttiaNet and ResNet1D architectures, including parameter count, computational cost, disk and VRAM utilization, as well as training and inference times. We note that the parameter count for AttiaNet (ranging from 593.5 k to 599.6 k) and ResNet1D (ranging from 6.94 M to 7.02 M) models grow slightly with the sampling rate because of the increasing feature map length at the output of the convolutions, thereby requiring more weights to connect to the fully connected layers. Nevertheless, the parameter count change across sampling rates has a negligible impact on disk utilization (less than 1 MB). Despite having approximately 12 times fewer parameters and disk utilization (599.6 K occupying 2.4 MB) compared to ResNet1D (7.021 M occupying 28.09 MB), the AttiaNet architecture yields significantly lower test MSE at 500 Hz, indicating the effectiveness of lightweight and streamlined architectures for ECG age estimation tasks. The substantial discrepancy in the parameter count across the two architectures, along with the large kernel size utilized in ResNet1D (k=17), makes ResNet1D 458× (at 500 Hz) more computationally expensive compared to the AttiaNet in terms of the number of floating point multiplicative and additive operations in the forward pass. Consequently, the practical application of the ResNet1D architecture is limited to machines with high computational capacity and modern GPUs. Furthermore, the ResNet1D necessitates of extensive GPU memory allocation for storing kernels and feature maps during forward and backward propagations, resulting in nearly 6 GB of VRAM utilization (21× higher). In contrast, AttiaNet training requires less than 300 MB VRAM, rendering it feasible to train on older GPUs with smaller VRAMs or even CPUs. The reduced computational cost and VRAM utilization enable AttiaNet to complete an epoch of over 16,000 ECG instances in just 4.1 s, while the ResNet1D architecture takes 245 s (at 500 Hz). Notably, the training of ResNet1D using tenfold cross-validation (200 epochs per fold) on T4 GPUs (power rating of 70 Watts) consumes 136 h and 9,527 watt-hours of energy, whereas training AttiaNet takes 2.27 h and consumes 160 watt-hours of energy (60× less). Hence, AttiaNet emerges as the sustainable choice for ECG age estimation. Moreover, the AttiaNet architecture demonstrates up to 2.7× faster performance at 100 Hz and 5× faster at 500 Hz compared to ResNet1D when performing test set inference on a GPU. The gap in inference times widens further on the CPU, with AttiaNet being 41× faster at 100 Hz and 76× faster at 500 Hz relative to the ResNet1D model.

**Table 2:** A tabular summary of training time per epoch, inference times for test set, disk and VRAM utilization, and computational cost of AttiaNet across different sampling rates.

Sampling Rate (Hz)	Training time (s)	Inference GPU (s)	Inference CPU (s)	Parameter Count	Disk Utilization (MB)	Forward/Backward Pass Memory (MB)	Estimate VRAM Usage w/Input (MB)	Multiplications-additions (G)
100	1.71	0.10	0.27	593,505	2.37	63.01	70.11	0.35
200	2.97	0.11	0.40	595,041	2.38	113.15	124.97	0.66
300	2.84	0.13	0.51	596,577	2.39	163.28	179.83	0.96
400	3.17	0.18	0.64	598,113	2.39	213.42	234.69	1.26
500	4.10	0.25	0.82	599,649	2.40	263.55	289.55	1.57

**Table 3:** A tabular synopsis outlining the training duration per epoch, inference times for test set, disk and VRAM consumption, as well as the computational expenses incurred by ResNet1D across various sampling rates.

Sampling Rate (Hz)	Training time (s)	Inference GPU (s)	Inference CPU (s)	Parameter Count	Disk Utilization (MB)	Forward/Backward Pass Memory (MB)	Estimate VRAM Usage w/Input (MB)	Multiplications-additions (G)
100	51	0.27	11.17	6,940,065	27.76	1,210.32	1,242.80	143.97
200	109	0.49	23.34	6,960,545	27.84	2,420.64	2,457.92	287.94
300	175	0.71	36.65	6,981,025	27.92	3,630.96	3,673.04	431.91
400	195	1.03	48.72	7,001,505	28.01	4,841.28	4,888.16	575.88
500	245	1.28	62.38	7,021,985	28.09	6,051.60	6,103.28	719.85

**Figure 4:**
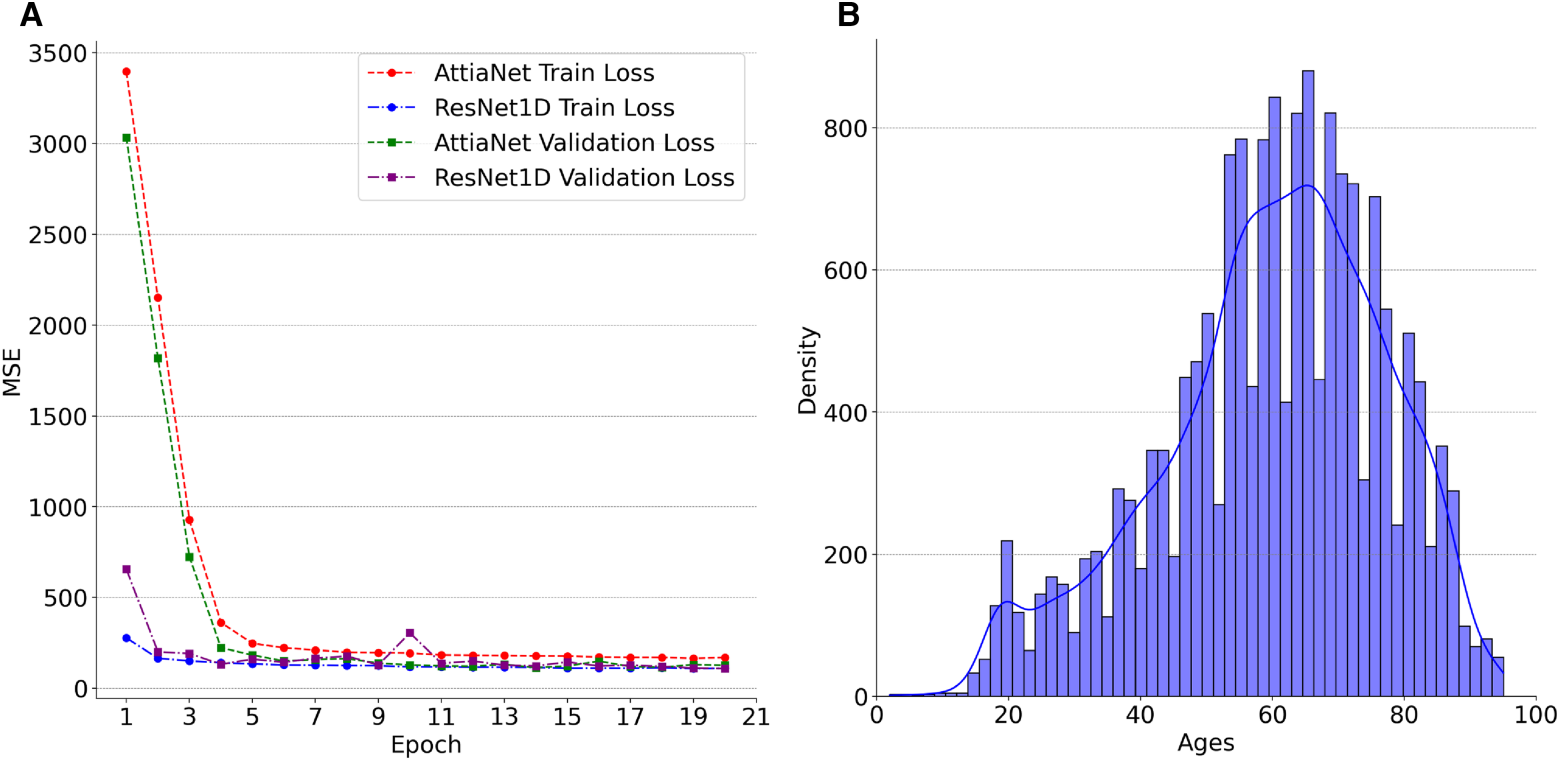
(**A**) Trends in the losses across the first twenty epochs of AttiaNet@500 Hz and ResNet1D@300 Hz for the first cross-validation fold. (**B**) Density plot of Ages in the training set for the first cross-validation fold.

We plot the MSE curves of the best-performing AttiaNet@500 Hz and ResNet1D@300 Hz architectures to investigate the differences in the learning ability between the two different neural network architectures. Additionally, we examine the chronological age distribution of the train set for the first fold of the tenfold cross-validation process to deduce meaningful insights from the test set inference. Specifically, Figure 4A highlights that the ResNet1D architecture achieves sub-500 train and validation MSE as early as the second epoch, indicating its higher capacity and ability to learn rapidly due to the large parameter count. In contrast, the AttiaNet architecture requires up to five epochs to attain a comparable MSE for the training and validation sets. Notably, the MSE curves for AttiaNet exhibit a smoother trajectory than those of ResNet1D. This smoother behavior can be attributed to the smaller parameter count of AttiaNet, which mitigates the inclination to overfit the training data, thereby facilitating a more stable training process. Analysis of Figure 4B reveals that the training set distribution is centered around 60 years, with most subjects between 40 and 80 years in the PTB-XL dataset. The train set distribution is left-skewed because of the presence of a few subjects in the range of 20 and 40 years. Corresponding to the train set, we observe that the chronological age distribution of the test set for the first fold is 59.7±16.79 years, suggesting that the train and test share similar distributions. AttiaNet@500 Hz results in MAE of 7.80±6.53 years, with the ECG age distribution of 59.79±13.61 years. Similarly, ResNet1D@300 Hz results in an MAE of 7.88±6.91, with the ECG age distribution of 61.14±14.57 years. We can deduce that the mean of the chronological age and ECG age distributions overlap better for the AttiaNet inference relative to the ResNet1D. Nevertheless, ResNet1D architecture results in a broader distribution (i.e., standard deviation closer to the chronological age) than the distribution generated by AttiaNet. The overall lower variability of the ECG age distribution compared to the chronological age distribution is due to the unbalanced nature of the train set for the subjects between 20 and 40 years, biasing the models to predict ECG ages closer to the center of the distribution. Interestingly, the MAE errors achieved by the models on the PTB-XL dataset are better compared to the ResNet1D on CODE-15% (53), ELSA-Brasil (54), and SaMi-Trop (55), which yielded MAE values of 8.38±7.00, 8.44±6.19, and 10.04±7.76 (45). Moreover, the MAE values are also similar to those reported by Attia et al. (37) for the Mayo Clinic ECG dataset (6.9±5.6) with the AttiaNet model. Altogether, the superior performance of the AttiaNet model over the ResNet1D architecture, while providing benefits in terms of computational complexity, disk and memory utilization, power consumption, and inference time, highlights the potential of neural networks for ECG age estimation on low-power mobile devices deployable on old clinical machines to smartwatches.

### Impact of signal duration

Figure 3B displays the line plots corresponding to the train and test MSE for the AttiaNet and ResNet1D model for durations ranging from 2 to 10 s. For the AttiaNet architecture, we observe a significant increase in the MSE (6.5% decrease) as the signal length decreases from 10 to 8 s. The MSE plateaus as the signal length decreases to 6 s and then increases linearly with further reduction in signal duration to 4 (4.1% decrease) and 2 (6.8% decrease) seconds. Altogether, the test MSE increases significantly from 102.09 to 121.96 (19.6% increase) as the signal length decreases from 10 to 2 s, indicating that signal duration is determining parameter for the AttiaNet architecture. Interestingly, the test set performance of the ResNet1D architecture remains unchanged with the initial changes in the signal duration. The test MSE for this architecture hovers around 110 as the signal duration decreases from 10 to 6 s. As the signal duration decreases to 4 s, the test MSE increases to 115.43 (4.7% increase) and continues to plateau for the 2 s signal length, indicating that 6 s signal length is the elbow threshold for the large neural networks for ECG age estimation. Overall, the ResNet1D architecture experiences a 4.7% increase in the test set MSE relative to 19.6% of the AttiaNet architecture, indicating that high parameter count and sophisticated network modules can assist networks in estimating ECG with minimal degradation (i.e., increased robustness) in performance with shorter-length ECG signals. These observations pertaining to the performance of the AttiaNet and ResNet1D architectures are in line with the law of robustness proved by Bubeck et al. (56), which states that the over-parametrization (e.g., high parameter count of ResNet1D) is necessary for smooth interpolation of the data and effective generalization. Moreover, we note that the test set MSE nearly overlaps for both the architectures for the intermediate signal durations (i.e., 4–8 s), emphasizing the potential of lightweight neural networks for ECG age estimation given sufficient temporal information.

The computational costs, disk and VRAM utilization, and inference times presented in Tables 2 and 3 hold relevance both for specific sampling rates (i.e., 100-500 HZ) and for different signal lengths (i.e., 2–10 s). ECG signals with a duration of 2 to 10 s, sampled at 500 Hz, encompass data points ranging from 1,024 to 5,120, which is equivalent to the 10-s signal with sampling rates varying in the range 100–500 Hz. For the AttiaNet architecture, 10-s ECG signal results in the lowest test MSE, implying that the model requires the storage of high-fidelity (i.e., 500 Hz) full-length (i.e., 10-s) ECG signal for precise ECG age estimation but provides computational savings, faster inference time, and lower VRAM utilization. Thus, AttiaNet could be deployed on mobile devices, clinical computers, and ECG machines with low computational capacity in developing countries, refugee camps, and war-prone regions. In contrast, ResNet1D architecture performs best with a 6-s ECG signal, thereby providing disk space savings in storing the ECG signals (i.e., 3,072 data points instead of 5,120) but having several times higher computational costs, VRAM utilization, and inference times. Consequently, ResNet1D can only be deployed on powerful computers with high computational capacity and dedicated GPUs while allowing minimal disk utilization.

**Figure 5:**
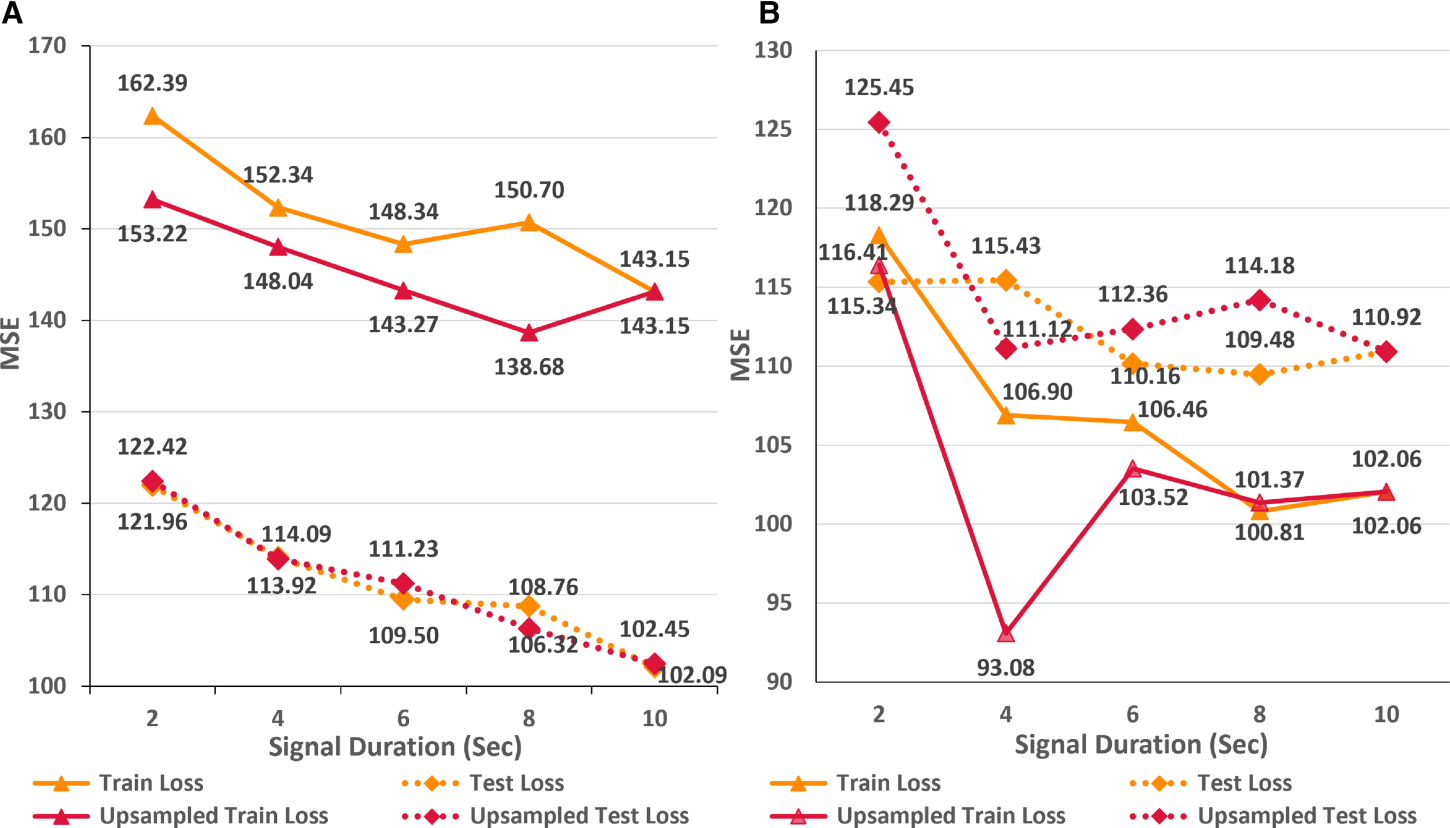
Comparison of train and test set performance for different durations of native and upsampled ECG signals for AttiaNet (**A**) and ResNet1D (**B**) architectures.

As an alternative approach, we hypothesize that upsampling the shorter ECG signals to encompass an equivalent number of data points as in a 10-s signal at a sampling rate of 500 Hz can compensate for the lack of temporal information. In order to test this proposition, we trained the AttiaNet and ResNet1D architectures with shorter signals (i.e., duration ranging from 2 to 8 s) upsampled to 5,120 data points. Figure 5 highlights the performance of AttiaNet and ResNet1D architectures at different signal durations at native and upsampled sampling rates. We observe that upsampling the shorter signal improves the train MSE for both architectures, indicating that higher signal density improves the memorization on the train set. For the AttiaNet architecture, the test MSE line with upsampled signal closely follows the one with a 500 Hz sampling rate, with slight deviations at 6 and 8 s signal lengths, suggesting a lack of improvement in generalizability. Interestingly, we observe a slight degradation in model performance with the ResNet1D architecture at shorter signal durations due to the information bottleneck created at the output neuron from larger feature maps generated from signals with 5,120 data points at the final convolution layer. At signal lengths of 4 s, ResNet1D achieves a much better train MSE minima, resulting in a slight improvement in test MSE over the signal sampled at 500 Hz. However, this phenomenon is an anomaly and is not observed in other signal durations, leading to the conclusion that upsampling short-duration signals does not improve the generalizability of the ECG age estimation networks.

### Impact of data augmentation

**Figure 6:**
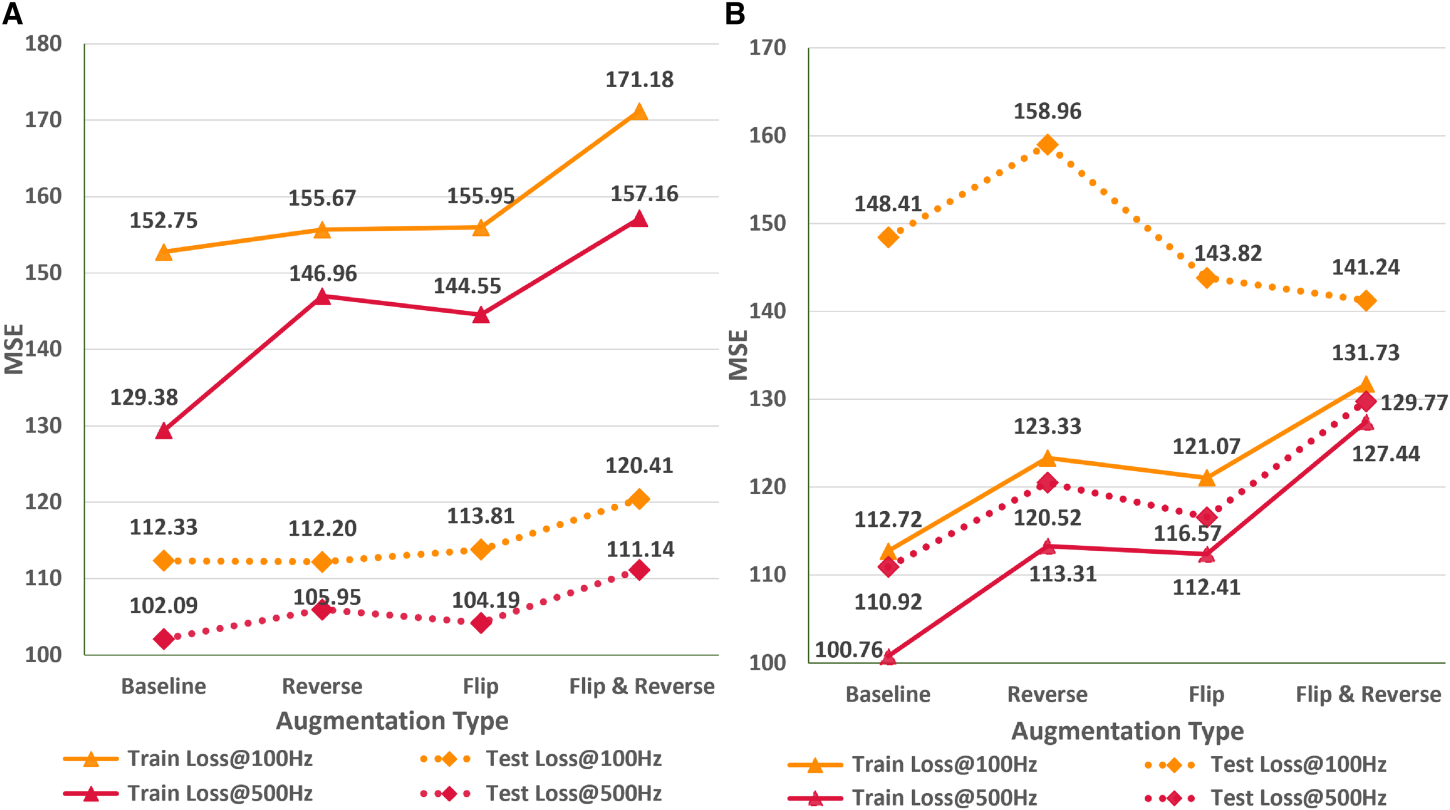
Impact of flip, reverse, and flip&reverse augmentation schemes on the performance of the AttiaNet (**A**) and ResNet1D architectures (**B**) at 100 and 500 Hz.

In this empirical study, we analyze the impact of different ECG signal augmentation schemes on the neural network’s performance for the ECG age estimation task. An effective augmentation scheme can allow neural networks to reduce the extent of overfitting and improve the generalizability of the unseen data. Figure 6 shows the variations in train and test MSE for the AttiaNet and ResNets1D architecture for two sampling rates across different simple augmentation schemes. Analysis of the increasing train MSE for both architectures suggests that augmentation schemes make the training routine challenging for the network by increasing variability in the batches at random. Specifically, the combination of flip and reverse augmentations significantly increases the dataset complexity, resulting in a higher train MSE relative to the baseline. Figure 6A indicates that the test set generalizability for the AttiaNet architecture does not improve with the flip or reverse augmentation application. Interestingly, the combination of flip and reverse results in an increase in MSE. Given that the network learns to identify anomalies throughout the signal, reversing the temporal information does not create a new pattern from the signal. Similarly, flipping the signal can be handled by adjusting the signs of the individual kernel weights. Nevertheless, the complexity of training increases due to the arbitrary application of the augmentation on the batches. Figure 6B reveals that the test MSE of ResNet1D follows a similar trend as the AttiaNet at 500 Hz. In this case, we note that flip, flip&reverse augmentations appear to improve the performance of the ResNet1D at 100 Hz. This phenomenon can be explained due to heavy overfitting of the ResNet1D at 100 Hz, which decreases with the increased complexity of the dataset with flip & reverse augmentation. Altogether, based on the proposed metrics and testing data sets, simple augmentation schemes like flipping and reversing signals do not seem to improve the accuracy capability of the network to estimate ECG age

### Impact of signal corruption

**Figure 7:**
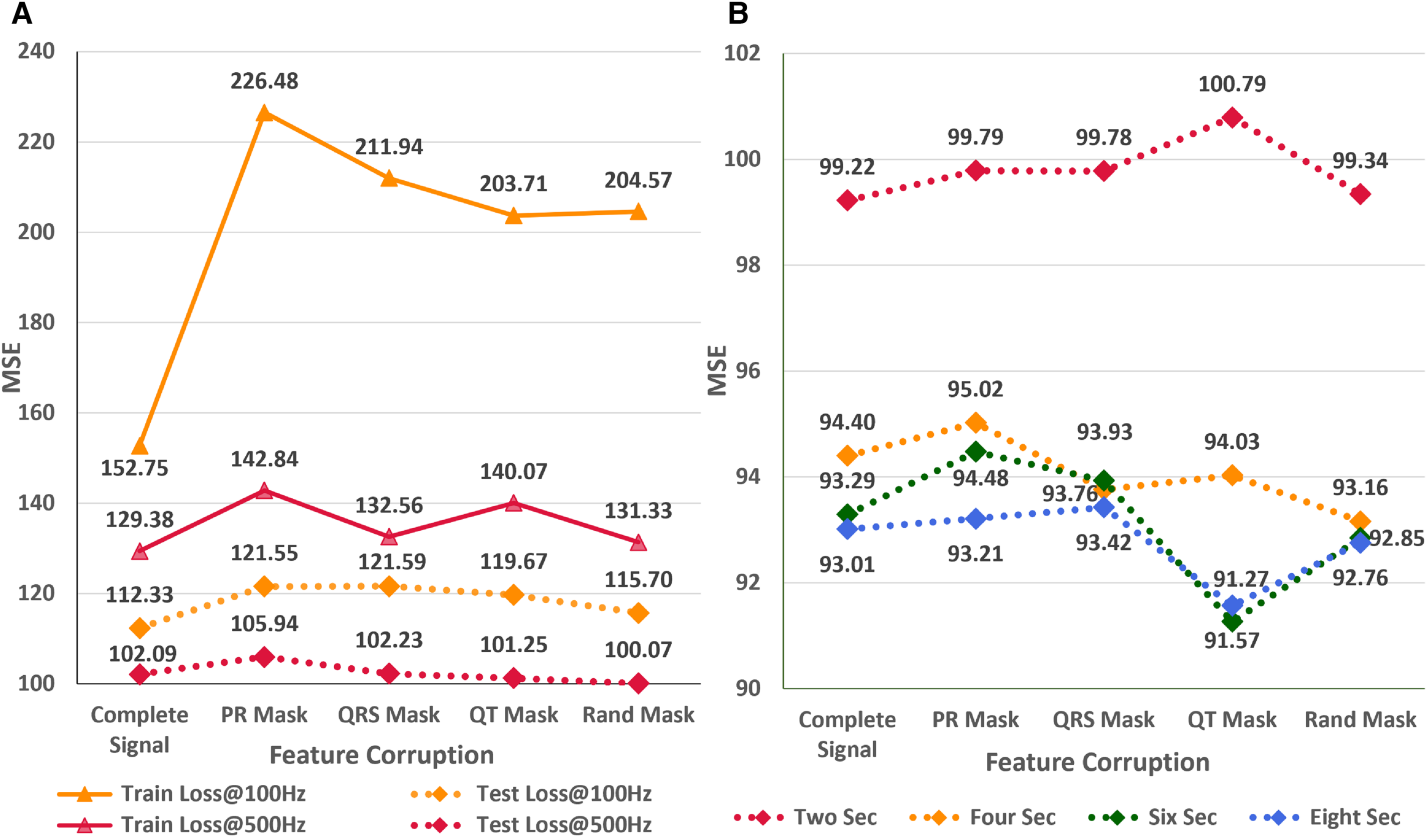
(**A**) Impact of different signal corruption schemes on the performance of the AttiaNet architecture at 100 and 500 Hz (**B**) Feasibility of random crop augmentation to overcome the corruption in ECG signals.

In the subsequent study, we analyze the effect of systematic and random signal corruption of some leads due to faults in the ECG machines or loose placement of electrodes. Figure 7A shows the performance of AttiaNet architecture under different signal corruption schemes. The training error of the network increases significantly at 100 HZ relative to 500 Hz because of the lower information density. Specifically, the feature corruption at 100 Hz leads to higher information loss because the remaining signal has a lower signal density. At 100 Hz, the test set MSE also increases to 121.59 (8.4% increase) with the QRS corruption and similar values with PR and QT corruptions. Interestingly, the degradation in the performance is least with the corruption of the signal at random locations, indicating that systematic loss of ECG morphological features impacts the network performance severely relative to arbitrary information loss. Surprisingly, at 500 Hz, most signal corruption schemes result in similar or improved performance in terms of the MSE. This observation can be explained by the higher signal density in the remaining portion of the signal that the network can utilize to estimate the age. Furthermore, arbitrary corruption of leads decreases the dependence of the network on specific leads. Consequently, signal corruption at higher sampling rates plays the role of an implicit dropout scheme at the data level, forcing the network to extract relevant information from all signal segments.

Finally, we implement a random crop augmentation scheme to improve the network’s performance for the ECG age estimation while using shorter-length signals. We restrict our experiments to AttiaNet due to the computationally heavy and time-consuming nature of ResNet1D architecture. Figure 7B show the performance of the AttiaNet architecture at 500 Hz, which is trained with random crops of the fixed window length ECG signal. Analyzing the results of the uncorrupted signal (i.e., complete signal) reveals that random crop enhances the performance across different window lengths (i.e., ranging from two to eight seconds). Maximum performance improvement is observed for 8-s random crops with an MSE of 93.01 (8.8% improvement), followed by the shorter window lengths. This increased performance of AttiaNet with random crops is because the network is fed with a slightly different portion of the original 10-s signal at every epoch, thereby forcing the network to extract relevant information depending on the portion of the signal, resulting in improved generalizability. Even the network trained with a 2-s random crop outperforms (99.22 MSE, 2.8% improvement) the best results of AttiaNet trained with the 10-s signal. These results indicate that random crop enables the network to provide improved performance with shorter signals (5 times disk space saving with 2-s signals) and reduced computational cost during the inference stage. Figure 7B also validates that the performance uplift provided by the random crop is invariant to the systematic and random corruptions in the ECG signal. Overall, the random crop is a robust ECG augmentation scheme that improves performance with complete and corrupted ECG signals.

### Impact of transfer learning

In this empirical study, we explore the applicability of existing networks trained on a specific ethnic population for accurately estimating the age of ECG signals from a different ethnic group. Additionally, we investigate the effectiveness of transfer learning and finetuning techniques in enabling neural networks to generalize across ethnic groups, particularly in scenarios of limited data availability. To conduct the experiments, we utilize the ResNet1D@400 Hz architecture since pre-trained weights for the AttiaNet architecture on the Mayo Clinic dataset are not accessible. Evaluation of ResNet1D@400 Hz across the ten test folds with pre-trained weights results in the test MSE of 154.2, which is significantly higher than the 104.6 (47.4% increase) achieved from scratch training on the PTB-XL dataset. These findings suggest that using a pre-trained network based on the Brazilian population may not yield precise ECG age estimation for individuals belonging to a German ethnic group.

**Figure 8:**
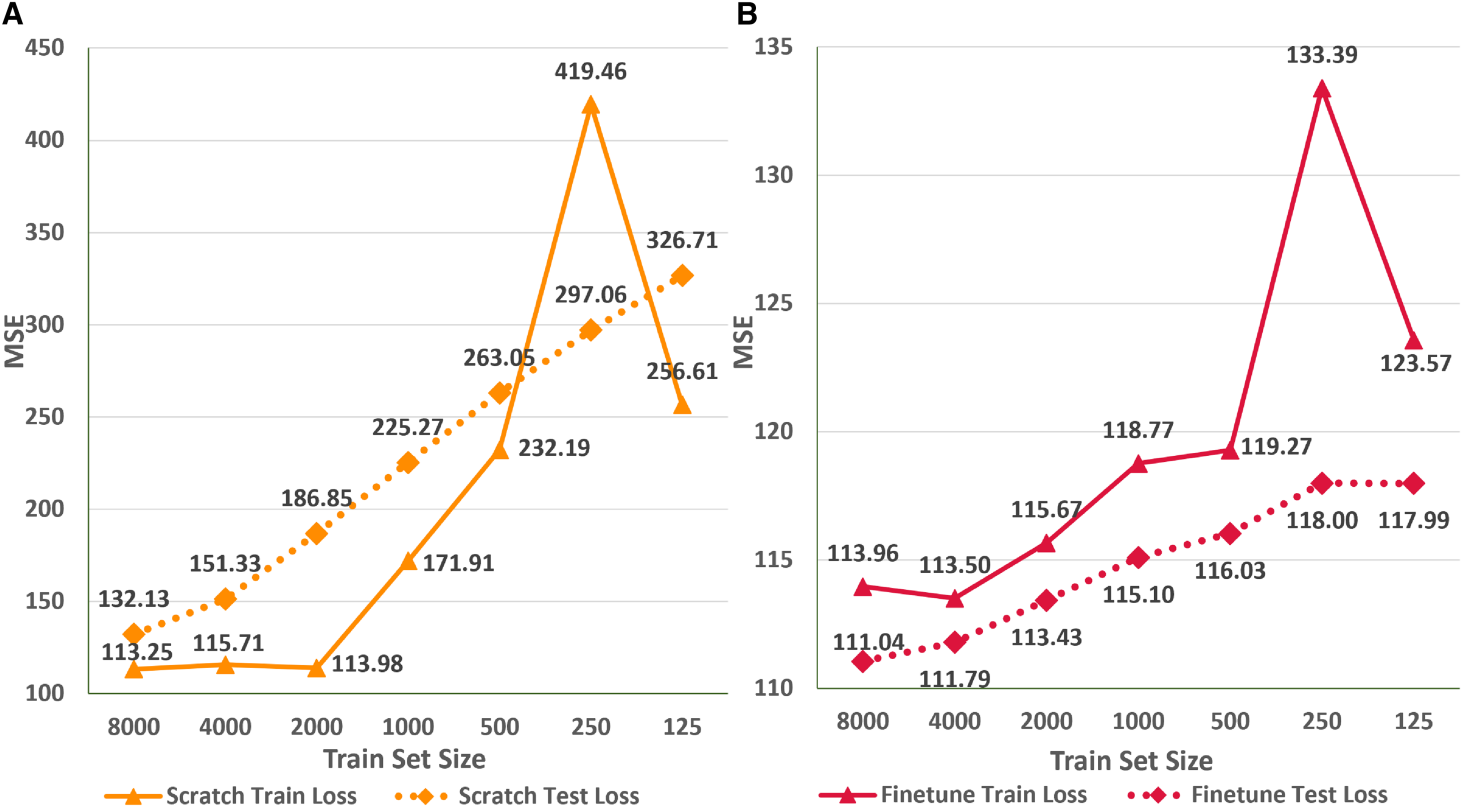
Comparison of train and test set performance for different train set sizes for the ResNet1D@400Hz with finetuning (**A**) and scratch training (**B**).

Figure 8 depicts the variation in the train and test MSE of the ResNet1D architecture at 400 Hz during scratch training (Figure 8A) and fine tuning (Figure 8B) with different sizes of training sets to simulate scenarios where data availability is limited. We observe that finetuning with 50% of the whole train set size (i.e., 8,000 instances) results in the test set MSE of 111.04 (6% increase over scratch training with the entire train set). To emphasize the advantages of finetuning, we retrain the ResNet1D@400 architecture by successively reducing the training set size by a factor of two. Interestingly, the test MSE increases to 115.1 at the train set size 1,000 and plateaus at 118 for small train set sizes of 125–250. Specifically, we decrease the train set size from 16,000 to 125 (128× decrease), and the test MSE increases from 104.6 to 118 (12.8% increase), highlighting the significance of fine tuning for the ECG age estimation task. As a direct implication of these results, neural networks trained on large ECG datasets (e.g., CODE cohort) can be fine tuned to estimate ECG age for the populations of developing countries, refugee camps with different ethnic groups, and war-prone regions, where large ECG datasets may not be available due to lack of digital transformation, infrastructure, and access to computing. In contrast, training the ResNet1D@400 Hz architecture from scratch with smaller training sets substantially increases the train and test MSE. For instance, decreasing the train set size from 16,000 to 8,000 leads to a test MSE increase from 104.6 to 132.13 (a 26.3% increase), further rising to 151.33 at 4,000 instances. As the train set size continues to decrease, the test MSE shows an approximate increase of 35 points. Consequently, there is a notable disparity in the test set MSE between fine tuning and scratch training, particularly for train set sizes below 2,000 instances. Figure 8 also illustrates a sharp increase in the train MSE at the train set size of 250 instances. The random selection of difficult ECG instances (i.e., containing high baseline wander and electrical equipment noise) instances out of the entire train set could cause this anomaly. Nevertheless, higher train set MSE does not influence the test MSE trend for scratch training and finetuning scenarios.

One of the primary concerns surrounding neural network-based methodologies is their environmental impact, particularly due to the substantial electrical power consumption during prolonged training periods (57, 58). In this context, we suggest that transfer learning and fine tuning offer can be additional factors to mitigate the training times and power consumption of large neural networks without significant performance degradation. Table 4 presents the training times per epoch and for ten-fold cross-validation, along with the net energy consumption for finetuning and scratch training scenarios across various train set sizes. We can deduce that scratch training takes 3.5× the time for the smaller train sets (e.g., 125 instances) and up to 5× the time for the large train sets (e.g., 1,000–8,000 instances) relative to finetuning. The significant discrepancy between the two training regimes arises because finetuning updates the weights of the final residual block and the fully connected layer, thereby training less than half the total parameters of the ResNet1D architecture (i.e., 3.22 M). Due to the finetuning regime’s shorter per epoch training times, a ten-fold cross-validation experiment for train set sizes of 1,000 or less can be completed in less than 50 min. Correspondingly, the scratch training regime can take over 4 h to train on 1,000 ECG instances. As a result, shorter finetuning times enable deep learning practitioners to retrain the model frequently, incorporating newly available ECG instances into the existing training set to account for any biases in the network. Another implication of the longer training times in the scratch training regime is significantly higher energy consumption. Specifically, a tenfold cross-validation experiment with 8,000 instances takes nearly a day to complete and consumes 1,688.48 Wh of energy. Recent studies have shown that energy consumption per household in the rural areas of developing countries like India is around 1,000 Wh per day (59), highlighting the power-hungry nature of deep learning methodologies. In comparison, the fine tuning regime for a dataset size of 8,000 consumes 345.92 Wh of energy (4.9× lesser energy consumption). As a crucial outcome of this experiment, we can infer that fine tuning large neural networks is an environmentally sustainable approach that can provide similar performance to the scratch training regime while utilizing smaller train set sizes and having shorter training times, allowing the usage of large networks for ECG age estimation in developing countries and war-prone regions with energy scarcity.

**Table 4:** Tabulation of training times per epoch and across 10-folds along with GPU energy utilization for the finetuning and scratch training scenarios.

Train Set Size	Finetune Epoch Time (s)	Finetune Total Time (min)	Fine Tune Power Consumption (Wh)	Scratch Training Epoch Time (s)	Scratch Training Total Time (min)	Scratch Training Power Consumption (Wh)
8,000	8.90	296.50	345.92	43.42	1,447.27	1,688.48
4,000	4.72	157.27	183.48	21.86	728.73	850.19
2,000	2.52	83.83	97.81	12.30	409.93	478.26
1,000	1.49	49.80	58.10	7.56	251.83	293.81
500	0.76	25.30	29.52	3.44	114.63	133.74
250	0.28	9.43	11.01	1.46	48.50	56.58
125	0.19	6.47	7.54	0.68	22.67	26.44

### Summary and impact of key outcomes

Determination of ECG acquisition parameters for acceptable ECG age estimation substantially impacts future research methodologies and practical utilization of ECG age in clinics. The outcome of the first experiment suggests that higher sampling rates ranging from 300 Hz to 500 Hz result in significantly better estimation of ECG age in terms of MSE for both neural networks. However, over-parametrized neural networks (e.g., ResNet1D) with high capacity can rapidly learn to estimate ECG age but may not result in the lowest MSE. Thus suggesting that lightweight networks (e.g., AttiaNet) can effectively estimate ECG age while providing computational and power savings (by up to 60×), highlighting the feasibility of ECG age estimation on ECG machines and personal smartwatches. The second investigation indicates that reducing signal duration can degrade neural network performance for ECG age estimation. Nevertheless, high-capacity (i.e., large number of parameters) neural networks (e.g., ResNet1D) are more robust to fluctuations in performance due to changes in ECG signal length. Interestingly, artificially upsampling shorter ECG signals improves the network’s performance on the train set without improving test set generalizability. Altogether, these experiments emphasize the significance of ECG acquisition parameters for ECG age estimation while providing the trade-off between computational cost and network performance when working with different acquisition parameters. Consequently, researchers can utilize ECG signals with a duration between 6 and 10 s captured at sampling rates between 300 and 500 Hz to develop models for ECG age, depending on their computational budget. In clinical practice, physicians can utilize existing ECG records with the above-mentioned acquisition parameters to accurately generate ECG age estimates with lightweight networks on low-computation mobile devices.

Transfer learning/fine tuning and ECG augmentation schemes can significantly improve the performance of neural networks for ECG age estimation. The third study validates that pre-trained neural networks for ECG age estimation of a different racial demographic may result in higher error (i.e., MSE) relative to scratch training due to the genetic underpinning of ECG and variations in clinical parameters. Nevertheless, finetuning pre-trained networks with substantially smaller train sets (i.e., several folds smaller) results in ECG estimation MSE within 10% of the scratch training. The finding suggests that ECG age can be made available in developing countries, war-prone regions, and refugee camps, where large-scale ECG datasets are not available due to the lack of digital infrastructure and financial resources. Furthermore, finetuning is environmentally sustainable for large neural networks, resulting in approximately five times less power consumption. Consequently, researchers should train their future novel architectures on large-scale datasets and share the weights with the open-source community so that the networks can be finetuned for different ethnicities while promoting sustainability. The fourth study shows that ECG augmentation schemes such as flipping and reversing do not improve network MSE for ECG age estimation. Additionally, systematic and random signal corruption can degrade the performance of networks at lower sampling rates while serving as data dropout schemes at higher sampling rates. Nevertheless, random cropping is a robust augmentation scheme that boosts networks’ ECG age estimation performance with shorter and corrupted ECG signals.

Even though the empirical study is thorough and comprehensive, it has some inherent limitations. The range of sampling rates evaluated in this study is between 100 and 500 Hz for the 10-s ECG signal. Evaluation of neural networks with higher sampling rates (e.g., 1,000 Hz) and longer signal duration (i.e., 20–30 s) would have provided additional insights to medical practitioners and enabled them to work with highest-end ECG configurations. Furthermore, the generalization study has been conducted for the Brazilian and German populations. Additional investigation is needed for generalization to African and South Asian populations. These limitations arise due to the lack of availability of ECG datasets for Asian and African populations with high sampling rates and long signal duration.

This empirical study focused on the impact of ECG acquisition parameters on the performance of neural networks for ECG age estimation. Specific thresholds for the acquisition parameters may vary from ECG age estimation to CVD diagnosis tasks because cardiac abnormalities reflect in individual waves of the ECG waveform. In future, we plan to extend this study to evaluate the impact of ECG acquisition on different CVDs, including arrhythmia and myocardial infarction. Additionally, we plan to modify the AttiaNet architecture to maximize its efficiency in terms of VRAM and disk utilization to deploy it on wearable devices such as Fitbits and smartwatches. Subsequently, we will evaluate the clinical utility of ECG age by utilizing the variations in ECG age extracted from routine ECG signals for hypertrophic cardiomyopathy in young athletes.

## Conclusion

In this paper, we conduct comprehensive experiments on cutting-edge neural network architectures for ECG age estimation to understand the significance of data acquisition parameters, data augmentation, and transfer learning. The study reveals that the performance (in terms of MSE, MAE) of the tested neural networks for ECG age estimation degrades with sampling rates below 200 Hz. This would suggest that a 300–500 Hz signal sampling frequency is necessary for accurate estimation. Expectedly, the performance of the tested networks degrades with the decrease in temporal information, especially with signals less than four seconds. Nevertheless, large neural networks are more robust to decrease in ECG signal duration. The study to analyze the performance of pre-trained and finetuned models revealed that transfer learning enables similar performance as scratch training with a fraction of the dataset size while being sustainable and environmentally friendly in terms of power consumption. Finally, we suggest the random crop data augmentation scheme that provides a 9% improvement in error relative to the best-performing model without ECG augmentation while using shorter duration signals.

## Data Availability

The dataset used for this empirical study is PTB-Xl52. The complete dataset is available for download on PhysioNet (https://physionet.org/content/ptb-xl/1.0.3/). The code for replicating the outcomes of the empirical study will be available on GitHub (https://github.com/ansariyusuf/ECG_Age_Empirical_Study).

## Footnotes

^1^https://github.com/klean2050/ecg-augmentations.

^2^https://pytorch.org/.

^3^https://github.com/antonior92/ecg-age-prediction.

## Glossary: Technical terms and their explanations.

One-dimensional CNNs: Neural networks that use one-dimensional convolutional layers to process sequential data for feature extraction and analysis.
Convolutional Layer: A neural network layer that applies convolution operations to input data, detecting patterns such as amplitude changes in signals.
Convolutional Kernels: Filters used in convolutional layers to scan input data for specific patterns, allowing for shared weights and efficient feature extraction.
Kernel Size: The size of the filter used in convolution operations, affecting the receptive field and the type of patterns detected.
Temporal Convolution: Convolution operations applied along the time dimension of sequential data to capture temporal patterns.
Spatial Convolution: Convolution operations applied to spatially arranged data to integrate information across different channels.
Residual Block: A neural network building block that includes skip connections to maintain information flow and mitigate vanishing gradients.
Skip Connection: A technique in residual networks where input is directly passed to subsequent layers, helping to preserve information and improve training.
Channel Dimension: The dimension along which multiple feature maps are stacked in a neural network, representing different aspects of the input signal.
Max-pooling: A pooling operation that selects the maximum value in a window, reducing the dimensionality of feature maps while preserving significant features.
ReLU Activation: A non-linear activation function (Rectified Linear Unit) that introduces non-linearity into the network, allowing it to model complex relationships.
Batch Normalization: A technique to normalize the inputs of each layer, improving training stability and generalization.
Dropout: A regularization technique where randomly selected neurons are ignored during training to prevent overfitting.
Multi-layer Perceptron: A type of neural network consisting of multiple fully connected layers used for final processing in classification/regression tasks.
Sampling Rate: The frequency at which data is sampled from a continuous signal, such as an ECG signal, impacting information density and signal quality.
Signal Duration: The length of the ECG signal, affecting the temporal information available for analysis and the computational resources required.
Data Augmentation: Techniques used to artificially expand the size of a dataset by creating modified versions of existing data instances, aiding in model training and generalization.
Transfer Learning: A machine learning technique where a model trained on one task is fine-tuned or adapted for use on a different but related task, often improving performance.
